# 

**Published:** 1888-07

**Authors:** 


### BOOKS AND PAMPHLETS RECEIVED.

OUTLINES OF VETERINARY PHARMACOLOGY. By Dr. Edward Vogel, editor of the "Archives of Veterinary Medicine." 1888. St. Petersburg.

BULLETIN No. 36. Agricultural College of Michigan, Vet. Dep't. Experimental work among cattle.

REPORT OF THE COUNCIL OF THE ZOOLOGICAL SOCIETY, for the year 1887. London.

SIXTEENTH ANNUAL REPORT OF THE BOARD OF DIRECTORS OF THE ZOOLOGICAL SOCIETY. Philadelphia.

THE CORNELL UNIVERSITY REGISTER, 1887-8. Ithaca.

TWENTY-SEVENTH ANNUAL REPORT OF THE CINCINNATI HOSPITAL, 1887. Cincinnati.

ON THE SKELETON IN THE GENUS STURNELLA, WITH OSTEOLOGICAL NOTES UPON OTHER NORTH AMERICAN ICTERIDÆ AND THE CORVIDÆ. By R. W. Shufeldt, M.D. Reprint from the Journal of Anatomy and Physiology, Vol. XXII.

OBSERVATIONS ON THE PTERYLOSIS OF CERTAIN PICIDÆ. By R. W. Shufeldt, M.D. Reprint from the Auk, April, 1888.

BIRD NOTES FROM LONG ISLAND, N. Y. By William Dutcher. Reprint from the Auk, Vol. V, No. 2.

ON EXERCISE FOR PREVENTION AND CURE OF DEFORMITIES. By. A. H. P. Leuf, M.D. Reprint Medical and Surgical Reporter, March 31, 1888.

Sopra un caso di Parziale Atrofia Degenerativa del Cuore da Lesione Nervosa. Memoria del Professore Vincenzo Colucci. Bologna. 1888.

### AMERICA (NORTH).

### CANADA.

Cap Rouge.—Le Naturaliste Canadien.
Montreal.—The Canadian Record of Science, Vol. III., No. 2.
Ottawa.—The Ottawa Naturalist.
Toronto.—Proceedings of the Canadian Institute, Vol. 5, Fas. 2. Annual Report of the Canadian Institute, session 1886-7, Medical Science.

### UNITED STATES.

Albany.—Medical Annals.
Augusta.—The Sanitary Inspector.
Baltimore.—Johns Hopkins University Circulars, Studies from the Biological Laboratory. Vol. IV., Nos. 3, 4.
Champaign.—Bullentin of Illinois State Laboratory of Natural History. Vol. II., Art. VII.
Charleston.—Proceedings Elliott Society, July '87 to March '88.
Chicago.—Weekly Drovers' Journal, National Live Stock Journal, Chicago Tribune.
Cincinnati.—Journal of the Cincinnati Society of Natural History. Vol. XI., No. 1.
Colorado —Proceedings of the Colorado Scientific Society. Vol. II., Part 2.
Detroit.—The Microscope.
Knoxville.—Agricultural Science.
Meriden.—Transactions of the Meriden Scientific Society. Vol. II.
New York.—American Veterinary Review, New York Medical Journal. Medical Analectic, Scientific American, Forest and Stream.
Philadelphia —Medical and Surgical Reporter, Therapeutic Gazette. American Naturalist, Reins and Whip, Quarterly Compendium of Medical Sciences.
Red-wing.—Public Health in Minnesota.
St. Louis.—Weekly Medical Review.
Wilmington.—Delaware Farm and Home.

### AMERICA (SOUTH).

### ARGENTINE REPUBLIC.

Buenos Ayres.—Anales del Museo Nacional, Anales de la Sociedad Cientifica Argentina. Tome XXV. Entrega, 1-3.

### ASIA.

### CHINA.

Shanghai.—Journal of the China Branch of the Royal Asiatic Society. Vol. XXII., No. 1-4.

INDIA.

BOMBAY.—Journal of the Natural History Society. Vol. III., No. 1.
MADRAS.—The Quarterly Journal of Veterinary Science.

### EUROPE.

### AUSTRIA.

HERMANNSTADT.—Verhandlungen und Mittheilungen des Siebenburgischen fur Naturwissenschaften. Jahrgang XXXVII.

VIENNA.—Annalen des K. K. Naturhistorischen Hofmuseums Band I–III. 1. Ornis. Internationale Zeitschrift fur die gesammte Ornithologie. Jahr III–IV., Heft. 1-2. Mittheilungen des Ornisthologischen Vereins in Wien, Oesterreichische Monatsschrift fur Thierheilkunde.

### BELGIUM.

BRUSSELS.—Annales de Medicine Veterinaire.
GHENT.—Annales et Bulletin de la Socéité de Medicine.

### DENMARK.

COPENHAGEN—Tisskrift fur Veterinærer.

### FRANCE.

LYONS.—Journal de Medicine Veterinaire et de Zootechnie.
PARIS.—Le Progress Medical, Recueil de Medicine Veterinaire.
TOULOUSE.—Revue Veterinaire.

### GERMANY.

AUGSBURG.—19-29–Bericht des Naturwissenschaftlichen Vereins fur S and H, 1867-87. Wochenschrift fur Thierheilkunde und Viehzucht.

BERLIN.—Archiv fur Wissenschaftliche und Praktische Thierheilkunde.

BONN.—Verhandlungen des Naturhistorischen Vereines der Preussischen Rheinlande Westfalens. Folge 5, Jahr 4.

BREMEN.—Abhandlungen von Naturwissenschaftlichen Vereinen. Bd X. Heft 1-2.

EMDEN.—Einundsiebenzigster Jahresbericht der Naturforschenden Gesellschaft.

FRANKFURT.—Der Zoologische Garten.

GIESSEN.—23 and 24 Bericht der Oberhessischen Gesellschaft fur Natur und Heilkunde.

KARLSRUHE.—Thierarztliche Mittheilungen.

KIEL.—Schriften fur Naturwissenschaftliche Vereine fur S. H. Band—III.-VII. Heft 1.

STUTTGART.—Repertorium der Thierheilkunde.

WURZBURG.—Sitzungsbericht der Physikalisch Medicinischen Gesellschaft 1887.

### GREAT BRITAIN.

EDINBURGH.—The Journal of Comparative Pathology and Therapeutics.
LONDON.—The Veterinary Journal, The Veterinarian.

### ITALY.

MILAN—La Clinica Veterinaria.
PAVIA.—Bolletino Scientifico Anno IX. No. 4.
PISA.—Giornale de Anat. Fisiol, e Patol. Degli Animali.
TURIN.—Bolletino dei Musei de Zoologia ed Anatomia Comparata della R. Universita. Vol. II.-III., Nos. 35-43.
UDINE.—Giornale di Veterinaria Militare.

### RUSSIA.

ST. PETERSBURG.—The Archives of Veterinary Science.

### SPAIN.

MADRID.—Gaceta Medico-Veterinaria.
VALENCIA.—La Cronica Medica.

### SWEDEN.

STOCKHOLM.—Tidskrift fur Veterinar-Medicine.

### SWITZERLAND.

BERN.—Mittheilungen der Naturforschenden Gesellschaft.
ZURICH.—Schweizer-Archiv fur Thierheilkunde.

